# *Parvimonas micra* forms a distinct bacterial network with oral pathobionts in colorectal cancer patients

**DOI:** 10.1186/s12967-024-05720-8

**Published:** 2024-10-17

**Authors:** Thyra Löwenmark, Linda Köhn, Therese Kellgren, William Rosenbaum, Vicky Bronnec, Anna Löfgren-Burström, Carl Zingmark, Pär Larsson, Michael Dahlberg, Bjoern O. Schroeder, Sun Nyunt Wai, Ingrid Ljuslinder, Sofia Edin, Richard Palmqvist

**Affiliations:** 1 Department of Medical Biosciences, Pathology, Umeå University, Umeå, Sweden; 2 Department of Mathematics and Mathematical Statistics, Umeå University, Umeå, Sweden; 3 Department of Diagnostics and Intervention, Umeå University, Umeå, Sweden; 4 Department of Molecular Biology, Umeå University, Umeå, Sweden

**Keywords:** Colorectal cancer, Intestinal microbiota, Oral pathobionts, *Parvimonas micra*, *Fusobacterium nucelatum*

## Abstract

**Background:**

Mounting evidence suggests a significant role of the gut microbiota in the development and progression of colorectal cancer (CRC). In particular, an over-representation of oral pathogens has been linked to CRC. The aim of this study was to further investigate the faecal microbial landscape of CRC patients, with a focus on the oral pathogens *Parvimonas micra* and *Fusobacterium nucleatum*.

**Methods:**

In this study, 16S rRNA sequencing was conducted using faecal samples from CRC patients (*n* = 275) and controls without pathological findings (*n* = 95).

**Results:**

We discovered a significant difference in microbial composition depending on tumour location and microsatellite instability (MSI) status, with *P. micra*, *F. nucleatum*, and *Peptostreptococcus stomatis* found to be more abundant in patients with MSI tumours. Moreover, *P. micra* and *F. nucleatum* were associated with a cluster of CRC-related bacteria including *Bacteroides fragilis* as well as with other oral pathogens such as *P. stomatis* and various *Porphyromonas* species. This cluster was distinctly different in the control group, suggesting its potential linkage with CRC.

**Conclusions:**

Our results suggest a similar distribution of several CRC-associated bacteria within CRC patients, underscoring the importance of considering the concomitant presence of bacterial species in studies investigating the mechanisms of CRC development and progression.

**Supplementary Information:**

The online version contains supplementary material available at 10.1186/s12967-024-05720-8.

## Introduction

Colorectal cancer (CRC) is a highly heterogeneous disease driven by a complex interplay among environmental, molecular, and genetic factors. Increasing evidence suggests that CRC is associated with intestinal dysbiosis, and large metagenomic studies have found a microbial shift in patients with CRC as compared with healthy individuals [1]. According to the driver–passenger theory, certain pro-oncogenic driver bacteria take part in the initial phase of tumourigenesis, possibly through modulating the tumour’s immune response and/or producing genotoxins that cause cell damage. As CRC progresses, the intestinal environment changes, creating new niches favouring colonisation by opportunistic bacteria. During this microbial shift, the initial driver bacteria are outcompeted by passenger bacteria that in turn can continue to promote tumour progression through inflammatory processes [2]. 

Interestingly, many of the bacterial species found to be increased in CRC patients are oral pathogens, including *Fusobacterium nucleatum* and *Parvimonas micra*. *F. nucleatum* is one of the best-studied bacteria in CRC and has been suggested to be a driver of CRC development [3–8]. It contributes to CRC progression through various mechanisms, including expression of the virulence factor FadA on the cell surface, which activates E-cadherin-mediated Wnt/β-catenin signalling [9]. *P. micra* is less studied but has also been associated with CRC [3, 5, 6, 10, 11], and can promote tumour formation through stimulating colonocyte proliferation and altering the Th17 immune response [11]. Other oral pathogens described to be associated with CRC include *Porphyromonas* spp., *Prevotella* spp., and *Peptostreptococcus* spp [12]. 

In our previous studies using targeted qPCR quantification of selected gut microbial taxa, we have found *P. micra* and *F. nucleatum* to be present in the same CRC patients, in both faecal and tumour tissue samples [13–15]. Moreover, both bacteria have been associated with tumours of the immune infiltrated MSI subtype, often found in right-sided CRCs [14, 15]. Interestingly, *P. micra* and *F. nucleatum* have been revealed to have a synergistic effect on biofilm formation, [16] a known feature of right-sided CRCs [17]. We therefore hypothesise that the associations found for these bacteria might be linked to a more complex interaction with the tumour microenvironment, involving a dynamic interplay among various bacterial species.

In this study, we therefore investigated the faecal microbial composition in CRC patients. We employed network analysis to identify clusters of bacteria associated with CRC, with a focus on *P. micra* and *F. nucleatum*. A better understanding of microbial co-occurrence in CRC could provide mechanistic insights into their role in tumourigenesis.

## Materials and methods

### Study cohort

The study is based on patients from the Faecal and Endoscopic Colorectal Study in Umeå (FECSU) and the Uppsala-Umeå Comprehensive Cancer Consortium (U-CAN) in Umeå, Sweden, both of which have been previously described in detail [18, 19]. 

In brief, the FECSU cohort comprises patients who underwent colonoscopy at the University Hospital in Umeå, Sweden, between the years 2008 and 2013. Indications for colonoscopy were gastrointestinal symptoms that indicated large bowel disease, visible blood in faeces, and/or haemoglobin in faeces (positive F-Hb). The colonoscopies were performed following standard routines at the endoscopy unit. Biopsies were taken when clinically relevant and classified by a pathologist according to clinical routine handling. In cases in which multiple neoplastic lesions were found, the most severe was used for classification. Out of 1997 patients invited to participate, 861 declined, resulting in 1136 participants. Of these, 39 were diagnosed with CRC, while the remaining patients were diagnosed with either dysplasia or had no neoplastic findings.

U-CAN longitudinally collects blood, tissue, radiological data, and clinical data over time from patients diagnosed with CRC in Umeå or Uppsala. From 2010 to 2014, additional stool samples were collected in Umeå. During this period, 684 CRC patients in Umeå were included in the project, and 260 of these patients provided a stool sample before initiating treatment. Subsequently, three patients opted to withdraw from participation, resulting in 257 CRC patients in the project.

### Study patients included

For the FECSU cohort, one CRC patient’s faecal sample was depleted, leaving faecal samples from 38 CRC patients available for further analysis. Few patients (*n* = 14) were included in both the FECSU and U-CAN cohorts. For these patients, the samples from the larger U-CAN cohort were excluded, resulting in 243 patients included from the U-CAN cohort. In addition, 100 control patients from the FECSU cohort, density matched for age and gender to the CRC cases, were also included in the study.

### Stool sample collection and storage

The stool sample collection and storage have previously been described [13, 18]. In brief, all stool samples were self-collected by the patients at home. For the FECSU cohort, patients were instructed to provide a stool sample before the pre-colonoscopy cleansing procedure. In the U-CAN cohort, stool samples were collected before initiating peri-operative antibiotic treatment or cancer therapy. The stool tubes used were pre-filled with 5 mL of RNAlater preservative buffer (Ambion, Austin, TX, USA). Following collection, all samples were stored at room temperature for a maximum of 7 days before centrifugation at 2000 rpm for 20 min. Subsequently, excess fluid was discarded and the samples were frozen at − 80 °C.

### Library construction and 16S ribosomal RNA gene sequencing

DNA was extracted from approximately 0.2 g stool samples using the QIAamp PowerFecal DNA Kit (Qiagen, Venlo, Netherlands) according to the manufacturer’s instructions. The Qubit dsDNA BR Assay Kit (Invitrogen, Carlsbad, CA, USA) was used to measure DNA concentration. The extracted DNA samples were sent to Novogene (Hong Kong, China) for sequencing on a NovaSeq platform. One of the most commonly targeted regions for microbial community and taxonomic studies, the 16S rRNA V3-V4 region, was chosen for analyses. The V3-V4 region is highly variable between different bacterial species and has highly conserved flanking regions, allowing efficient sequencing and sufficient discrimination between different taxa. Microbiota DNA was amplified using PCR with the bacterial 16S rRNA V3–V4 regions universal primer pair forward-CCTAYGGGRBGCASCAG and reverse-GGACTACNNGGGTATCTAAT. The sequencing produced 250 base pairs of paired-end raw reads.

### Sequencing data processing

Of the in total 281 CRC samples and 100 control samples, 275 (97.9%) CRC samples and 95 (95.0%) control samples passed all quality control steps from nucleic acid purification to 16S rRNA sequencing. Demultiplexed paired-end sequencing data were obtained as libraries per sample. Data were processed using the R statistical computing environment (version 4.02), and sample sequence inference was carried out with the DADA2 package (version 1.16). The DADA2 pipeline is commonly used today for analyses of microbial sequences. It includes a refined approach of error correction and resolves sequences to their exact biological sequences (ASVs), which provides higher resolution and better representation of the microbial diversity. In detail, quality profiles for the forward and reverse reads were generated using the plotQualityProfile function. Filtering involved setting the trunk length to 0 due to high-quality reads, and a q-score cutoff of 30 was applied. The merging of forward and reverse reads to generate amplicon sequence variants (ASV) was performed using the mergePairs function with default settings [20]. Taxonomy was subsequently assigned to the resulting ASVs using silva species assignment (version 138.1). ASVs represented by fewer than 50 reads within one sample were removed and the remaining ASVs were then postclustered based on co-occurrence, similarity, and abundance with the LULU algorithm [21]. The purpose of the LULU algorithm was to reduce the number of erroneous ASVs to achieve more realistic biodiversity metrics. By evaluating the co-occurrence patterns of ASVs among samples, LULU can identify ASVs that consistently satisfy criteria for being errors of more abundant ASVs and merges these highly similar sequences.

ASVs not assigned to any phylum or present in fewer than 10% of the samples following postclustering with LULU were removed. ASVs assigned to *F. nucleatum* did not cluster together in the LULU algorithm, likely due to the high genetic diversity of these bacteria. The taxonomy of *Fusobacterium* was recently revised with the subspp. *animalis* and *vincentii* being elevated to the rank of species [22]. These ASVs were therefore manually clustered together so as not to be removed in the filtering process and to ensure comparability with earlier studies. For ASVs that either could not be classified to species level, or where several ASVs with identical taxonomic classification persisted after Lulu curation, a running number was added to the ASV name. K-means clustering was then used to confirm that cancer stage IV did not stand out as a delimited cluster, so stage IV samples were continuously included in downstream analyses. In total, 367 ASVs remained for subsequent analyses.

### Molecular analyses

To perform molecular analyses, a 2–3-mm cube of fresh frozen tumour tissue was homogenised using the Precellys^®^ Soft Tissue Homogenizing CK14 Kit (Bertin Technologies, Rockville, MD, USA). DNA was extracted using the AllPrep DNA/RNA/miRNA Universal Kit (Qiagen, Hilden, Germany). For patients lacking fresh frozen tissue specimens, five sections (10 μm) of formalin-fixed paraffin-embedded (FFPE) tumour tissues were used instead. Fresh frozen tissues and FFPE tissues with less than 20% tumour cells were excluded. DNA was extracted using the AllPrep DNA/RNA FFPE kit (Qiagen, Hilden, Germany). The Qubit dsDNA BR Assay Kit (Invitrogen, Carlsbad, CA, USA) was used to measure DNA concentration.

The MSI Analysis System Version 1.2 (Promega, Madison, WI, USA) was used to determine MSI status, based on the analysis of the mononucleotide repeat markers BAT-25, BAT-26, NR-21, NR-24, and MONO-27 with the Peak Scanner™ Software v1.0 (Applied Biosystems, Foster City, CA, USA), as previously described [23]. Tumours with two or more altered markers were classified as MSI. Remaining tumours were classified as microsatellite stable (MSS). The digital droplet PCR (ddPCR; Bio-Rad Laboratories, Hercules, CA, USA) was used for analysis of the *BRAFV600E* mutation, as described previously [23]. For the *KRAS* mutation analysis, codons 12 and 13 were sequenced using Big Dye v.3.1 (Applied Biosystems, Foster City, CA, USA) and Sanger sequencing on a 3500xL Dx Genetic Analyzer (Applied Biosystems, Foster City, CA, USA). The primers and probes used for *BRAF* and *KRAS* mutation analyses have been described previously [23]. 

## Statistics

### Clinical and molecular characteristics

Statistical analyses were performed using IBM SPSS Statistics 28. For associations between categorial variables, the χ^2^ test was used.

### Alpha and beta diversity calculations

Alpha diversity Shannon, Simpson, Ace, and Chao1 indices were calculated with skbio on data that were rarefied using the R package vegan to the depth of the minimum sum of all ASVs (100 iterations). Mann–Whitney U (MWU) tests were used to evaluate differences in index means with scipy. Beta diversity using the avgdist function of vegan was estimated using Bray–Curtis distances on rarefied data (100 iterations) and principal coordinate analysis was performed with skbio. Permanova with 999 permutations (skbio) was used to test for differences in dispersion. Taxonomic relative abundance was computed and used in downstream analyses.

### Cancer and control classification

MWU tests were performed to test for differences in relative abundance. The mean and effect size were calculated with Cliff’s delta [24]. The eXtreme Gradient Boosting (XGBoost) classifier was trained using 1000 decision trees, with 80% of the data being used for training and 20% for testing [25]. To adjust for class imbalance, weighted XGBoost was used with the weights iterated to find the one that produced the best area under the curve. The XGBClassifier function of the python xgboost package was used.

### Faecal microbial composition in relation to clinicopathological and tumour molecular features

MWU tests were performed to test for differences in relative abundance. The mean and effect size were calculated using Cliff’s delta. In the MWU calculations for tumour location, the different locations were grouped as right colon versus left colon and rectum.

### Constructing faecal microbial composition co-expression networks

Pearson’s correlations were calculated between the ASVs in the cancer and control datasets, respectively, in the search for increasing or decreasing patterns between different ASVs, using the corr function of Pandas. If the absolute values of the correlations were larger than a cutoff, $\:\tau\:$, the elements were set to one, otherwise to zero, creating an adjacency matrix. The cutoff, $\:\tau\:$, was chosen through an iterative process so that the numbers of single nodes and small clusters were minimised in both matrices. This resulted in two adjacency matrices with a sparsity of 0.5%, including 672 and 646 edges for the cancer and control datasets, respectively. Faecal microbial co-expression networks were generated from the adjacency matrix using the walktrap algorithm as implemented in igraph [26]. 

To analyse the difference in abundance between bacteria identified at species level in the *P. micra* cancer cluster from the walktrap analysis, the raw counts values were transformed with centered log transformation (clr), using the decostand function in vegan (v. 2.6.6). Statistical testing on the clr adjusted values was performed using MWU, and the resulting *P* values were corrected using Holm’s method in the padj function in R (v. 4.4). Figures were created utilising the ggplot package in the tidyverse library (v. 2.0).

## Results

### The faecal bacterial composition of CRC patients

The faecal bacterial microbiomes of 275 study patients and 95 controls were assessed by sequencing of the V3-V4 region of the 16S rRNA gene. The clinical and molecular characteristics of the included study patients and controls can be found in Table 1. The sequencing data were subsequently processed by DADA2, post-clustered using the LULU algorithm, and ASVs present in 10% or more of the samples were included, resulting in a total of 367 ASVs.

**Table 1:** Clinical and molecular characteristics of the colorectal cancer study patients

	Cancer	Control	*p*-value
	*n* = 275	*n* = 95	
**Age (%)**			
≤59	45 (16.4)	15 (15.8)	0.745
60–69	103 (37.5)	33 (34.7)	
70–79	93 (33.8)	31 (32.6)	
≥80	34 (12.4)	16 (16.8)	
**Gender (%)**			
Female	111 (40.4)	41 (43.2)	0.633
Male	164 (59.6)	54 (56.8)	
**Location (%)**			
Right colon	61 (22.2)	NA	
Left colon	51 (18.5)	NA	
Rectum	163 (59.3)	NA	
**Stage (%)**			
I	50 (18.2)	NA	
II	95 (34.5)	NA	
III	77 (28.0)	NA	
IV	43 (15.6)	NA	
***BRAF *** **status** ***(%)***			
Wildtype	170 (82.5)	NA	
Mutated	36 (17.5)	NA	
***KRAS *** **status (%)**			
Wildtype	135 (66.5)	NA	
Mutated	68 (33.5)	NA	
**MSI status (%)**			
MSS	182 (88.3)	NA	
MSI	24 (11.7)	NA	

*X*^2^ tests were used to compare categorical variables

*F. nucleatum* is one of the best-studied bacteria in relation to CRC, with a higher abundance in CRC patients, and has in previous studies been associated with different important clinicopathological and molecular traits, including a worse prognosis [27], [28–30]. In this cohort, we found several ASVs annotated to *F. nucleatum*. However, these did not cluster together in the LULU algorithm, which could be due to different subspecies. Thus, all ASVs annotated to *F. nucleatum* were removed when filtering for ASVs found in fewer than 10% of the samples. *F. nucleatum* has previously had four known subspecies: *animalis*, *nucleatum*, *polymorphum*, and *vincentii*, with genetic differences. Very recently, *animalis* and *vincentii* were reclassified as separate species [22]. Using BLAST, all ASVs could be linked to the *F. nucleatum* species. However, we could not with certainty distinguish *F. nucleatum* from *F. animalis* or *F. vincentii.* Even though all subspecies have been found in CRC patients, studies at the subspecies level are scarce [31]. Nevertheless, the *F. nucleatum* species have proven to be highly interesting in the CRC context. Thus, in order not to lose important information, all ASVs annotated to *F. nucleatum* were clustered together and included in subsequent analyses.

**Fig. 1:**
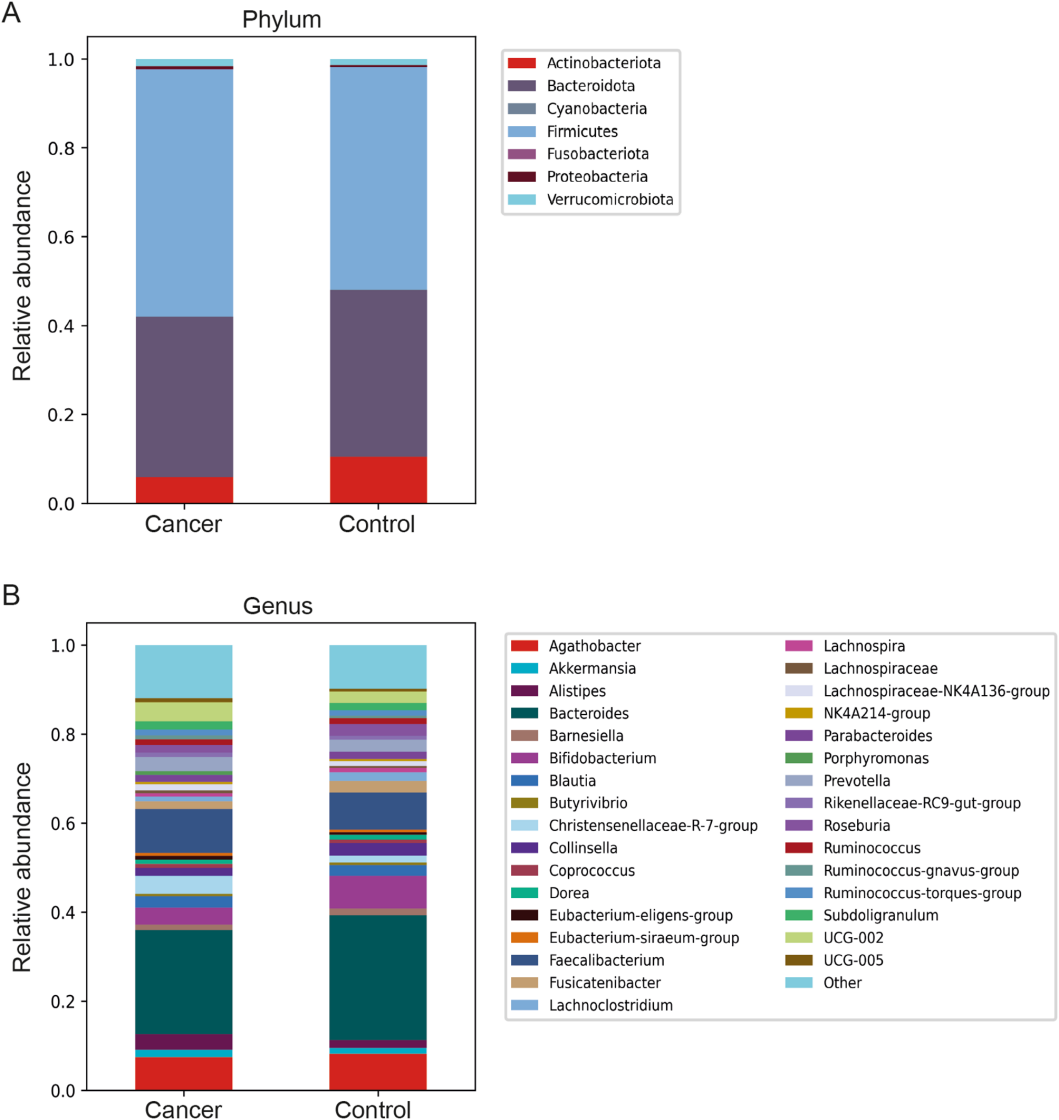
Taxonomic analysis of the faecal microbiomes of CRC patients and controls represented at the (a) phylum level and (b) genus level

The most abundant phyla in all samples were *Firmicutes*, *Bacteroidota*, *and Actinobacteria*, followed by *Verrucomicrobiota* and *Proteobacteria*, with a higher proportion of *Firmicutes* and a lower proportion of *Actinobacteria* and *Bacteroidota* found in CRC patients (Fig. 1a). The bacterial profile at the genus level is shown in Fig. 1b, displaying a lower proportion of *Agathobacter* (from the family *Lachnospiraceae*), *Bacteroides*, and *Bifidobacterium* in CRC and a higher proportion of *Alistipes*,* Faecalibacterium* and the *Christensenellaceae* R7 group (Fig. 1b).

**Fig. 2:**
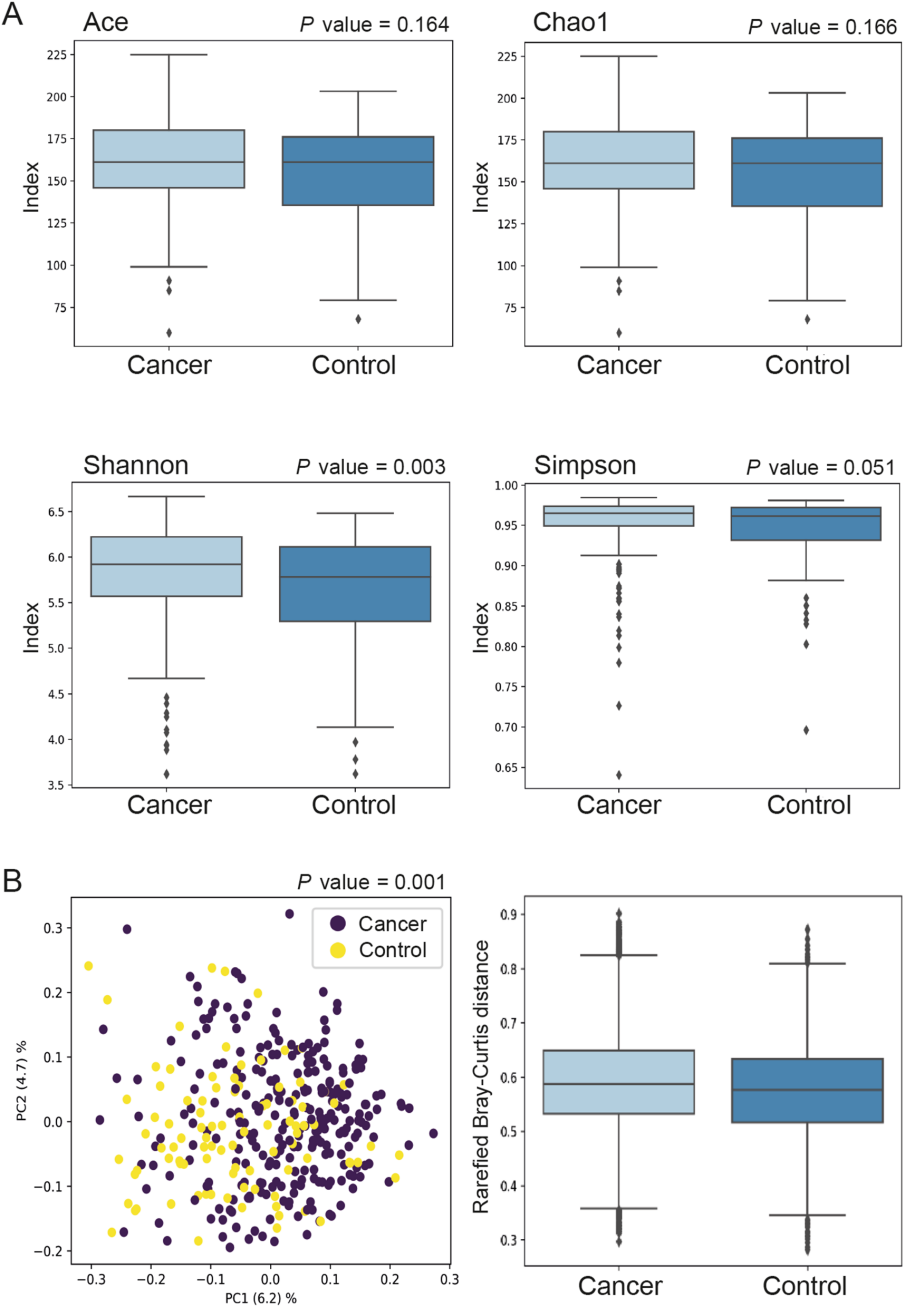
Intestinal bacterial richness and diversity in faecal samples of CRC patients and controls: **(a)** alpha diversity analyses based on Ace, Chao1, Shannon, and Simpson indices; **(b)** beta diversity analysis using Bray–Curtis distances

To determine the shifts in faecal bacterial communities between cancer patients and controls, alpha and beta diversities were analysed (Fig. 2). The Shannon (*p* = 0.003) index, but not the Ace (*p* = 0.164) or Chao1 (*p* = 0.166) index, showed a significant difference in alpha diversity between CRC patients and controls, with a higher microbial diversity found in faeces from CRC patients (Fig. 2a). The Simpsons index displayed borderline significance (*p* = 0.051) (Fig. 2a). Since the Shannon and Simpson indices take both the species richness and evenness into account, the results indicate that there is a difference in the relative abundance of ASVs between cancer patients and controls and/or a difference in distribution, while the Ace and Chao1 measures indicate that there is no difference in the number of ASVs. The Bray–Curtis distance was used for beta diversity comparison of differences in community composition. A significant separation of clusters between faecal samples from CRC patients versus controls (*p* = 0.001) was found, indicating that the gut microbial composition of CRC patients significantly differed from that of controls (Fig. 2b).

To analyse the differences in bacteria between cancer patients and controls in more detail, MWU tests were applied to rank the top ASVs with the most significant differences in abundance between the two groups (Additional file 1: Figure S1). Top ASVs more abundant in CRC included the *Christensenellaceae* R7 group, various *Porphyromonas* spp., *Prevotella* spp., *Alistipes* spp., *Coprococcus catus*, *Peptostreptococcus stomatis*, and *P. micra* (Additional file 1: Figure S1).

**Fig. 3:**
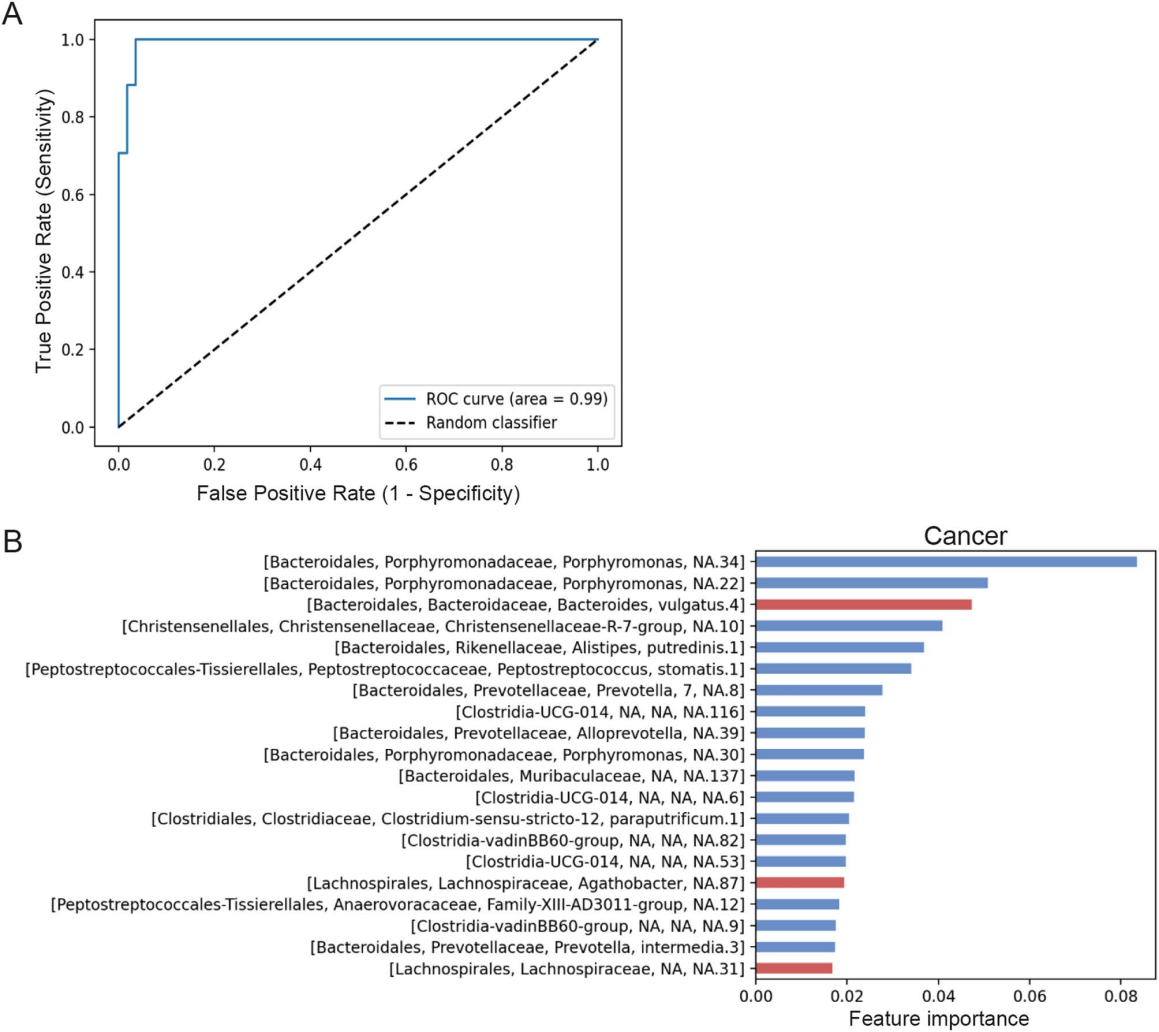
The XGBoost algorithm was used to determine the taxa most likely to reveal a difference between faecal samples of CRC patients and controls, as illustrated by the resulting **(a)** ROC-curve, and **(b)** the top 20 most important prediction taxa. Blue and red bars represent ASVs with a higher expression in patients and controls, respectively

Moreover, the XGBoost algorithm was used to predict the taxa most likely to reveal a difference in faeces between CRC patients and controls (Fig. 3). The area under the resulting ROC curve (AUC) for the model was 0.99 (Fig. 3a). The most important taxa for the prediction model, found to be more abundant in faeces from CRC patients, included various *Porphyromonas* spp., the *Christensenellaceae* R7 group, *Alistipes putredinis*, *P. stomatis*, bacteria form the class *Clostridia*, and *Prevotella* spp., whereas *Bacteroides vulgatus* was depleted in CRC patients (Fig. 3b). These taxa largely agree with previous findings regarding microbial segregation between CRC patients and healthy individuals. However, the importance of each taxon varies across datasets [4, 32–34]. 

### The faecal microbial composition differs between patients according to tumour location and MSI status

**Fig. 4:**
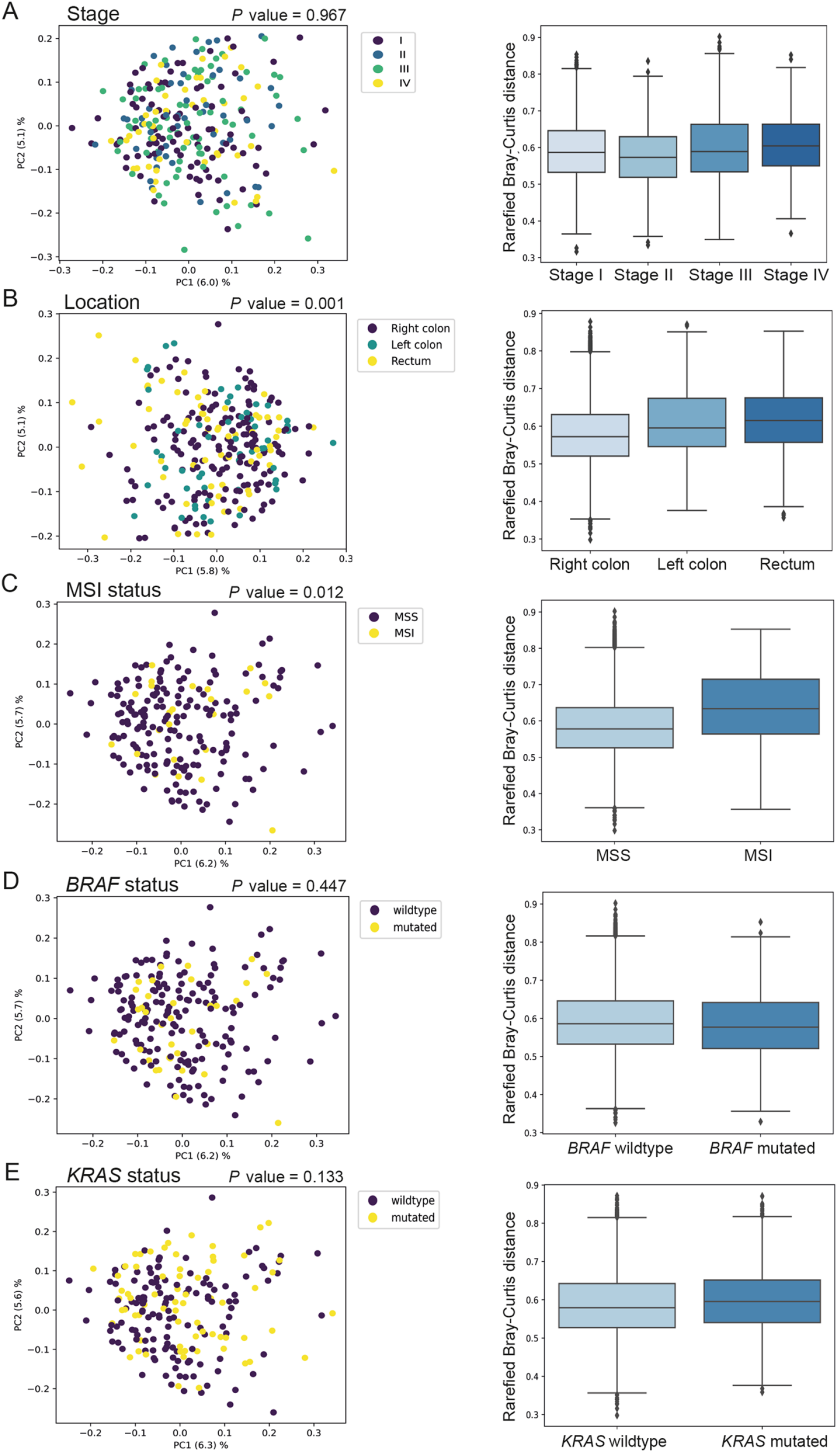
Beta diversity analysis using the Bray–Curtis distances of microbial species for **(a)** tumour stage, **(b)** tumour location, **(c)** MSI status, **(d) ***BRAF* mutation status, and **(e) ***KRAS* mutation status

To determine whether the composition of the faecal microbiome in CRC patients differs according to clinicopathological and tumour molecular features, beta diversity using Bray–Curtis distances was calculated based on tumour stage, tumour location, MSI status, and *KRAS* and *BRAF* mutation status (Fig. 4). Beta diversities were significantly different for tumour location (Fig. 4b) and MSI status (Fig. 4c). However, no difference in beta diversity was seen according to tumour stage or *BRAF* and *KRAS* mutation status (Fig. 4, a, d, and e).

**Fig. 5:**
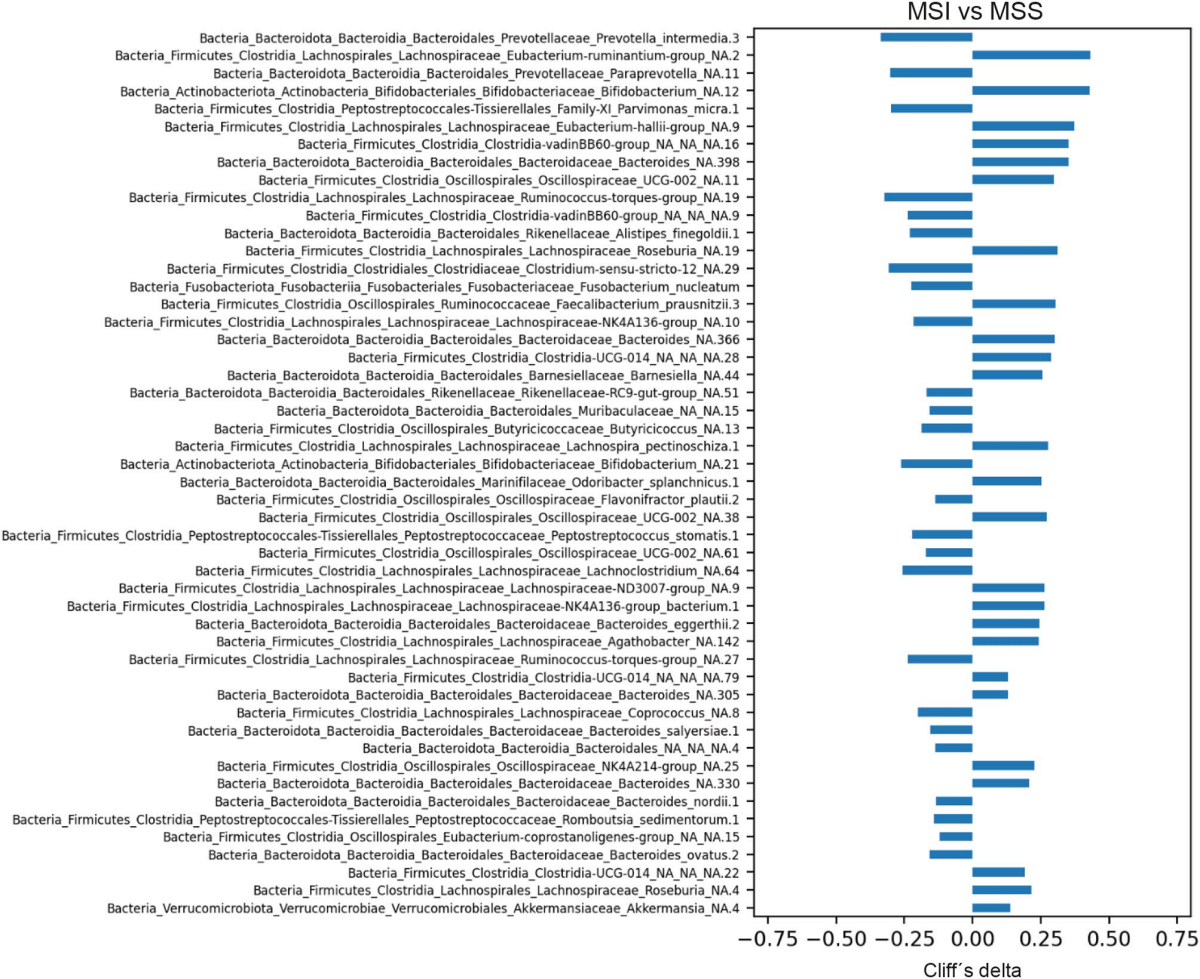
Top ASVs with the most significant differences in abundance between MSI and MSS tumours as determined by the MWU test. The ASVs were sorted according to p-value, showing the ASV with the lowest p-value at the top (nominal p-values were used). A negative Cliff’s delta indicates higher abundance in MSI tumours, whereas a positive Cliff’s delta indicates higher abundance in MSS tumours

Analyses using MWU tests were performed to evaluate differences in ASV abundance between MSI and MSS tumours. In the top 30 determinant taxa, ASVs more abundant in MSI tumours and annotated at the species level were *Prevotella intermedia*, *P. micra*, *Alistipes finegoldii*, *F. nucleatum*, *Flavonifractor plautii*, and *P. stomatis* (Fig. 5).

Since MSI tumours are more common in right-sided tumours and a significant difference in beta diversity was found for tumour location, analysis using the MWU test was subsequently conducted to compare taxa for the right colon versus the left colon and rectum (Additional file 2: Figure S2). However, no associations with the species found to be strongly associated with MSI tumours were found.

### Network analysis of faecal microbial composition reveals a cluster of CRC-associated bacteria

**Fig. 6:**
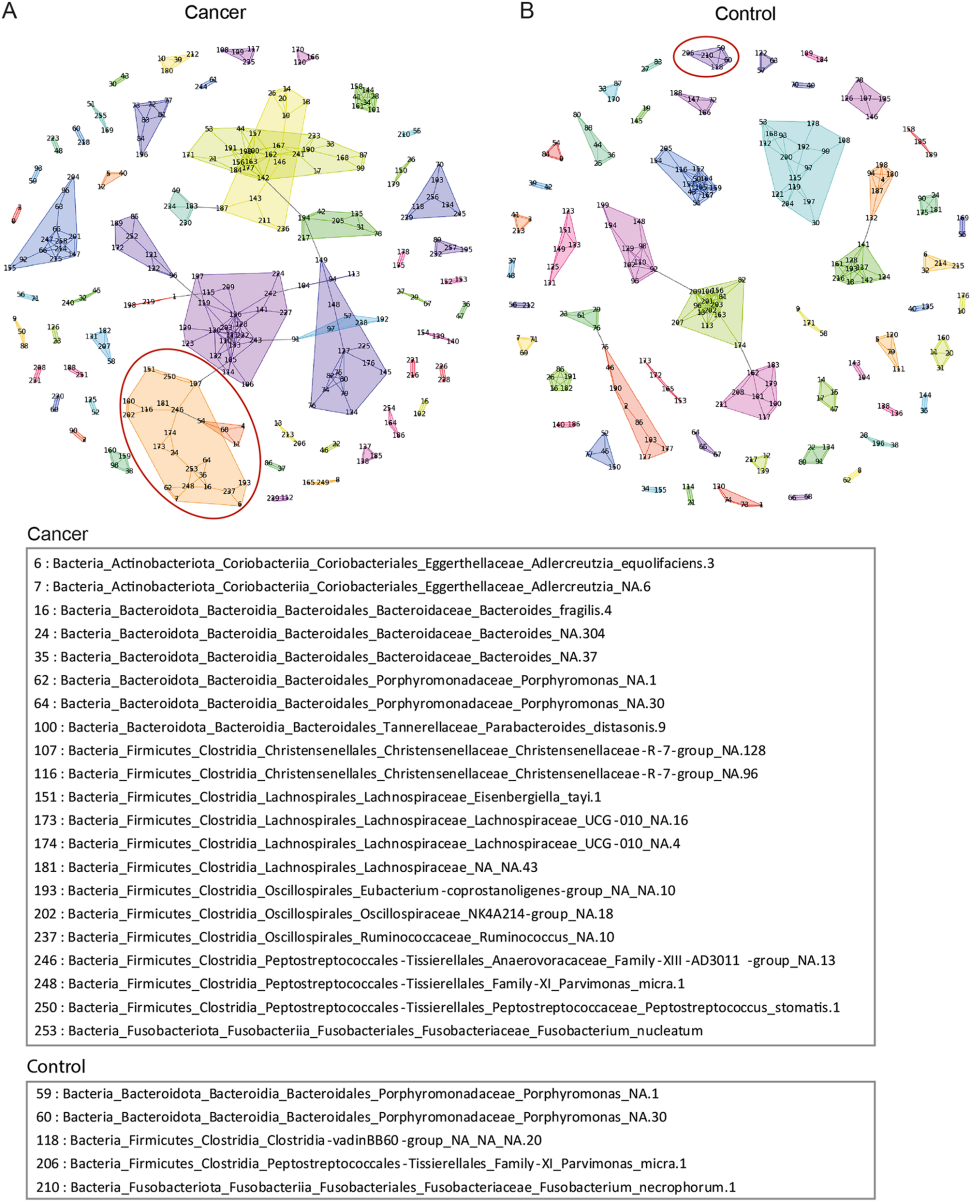
Walktrap network analysis: **(a)** cluster containing *P. micra* in faeces of CRC patients; **(b)** cluster containing *P. micra* in faeces of controls

**Fig. 7:**
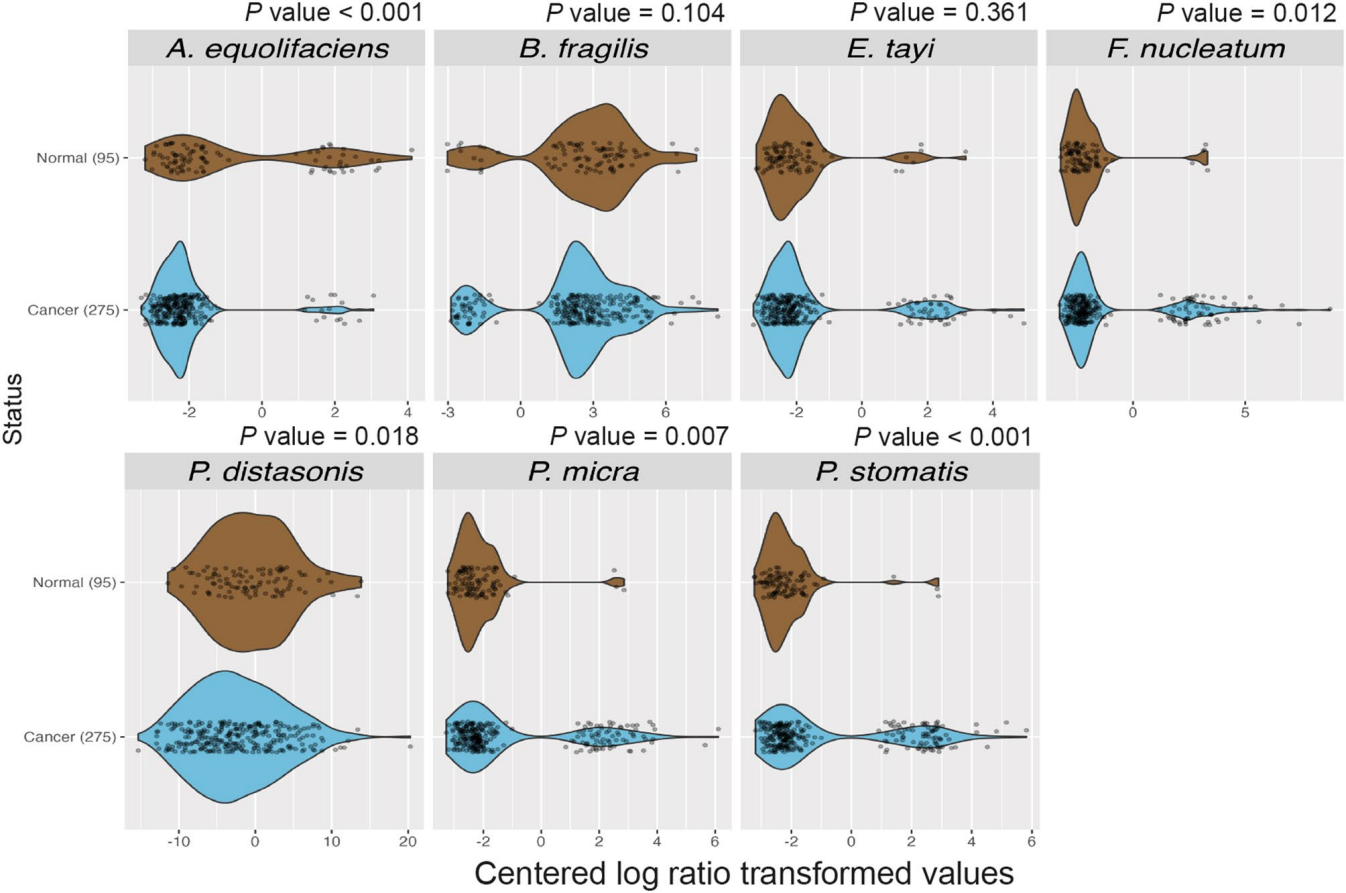
Violin and scatter plots for the distribution of bacterial counts for each bacterium at species level identified within the *P. micra* cluster. The count values were centered log ratio transformed using the vegan package (v. 2.6). MWU tests were used to analyse differences between cancers and controls for each bacterium. *P* values were corrected for multiple comparisons using the Holm method. A *P* value < 0.05 was considered statistically significant

To investigate the relationships between different bacteria in the faeces of CRC patients and controls, we next studied the correlations between different taxa using walktrap network analysis (Fig. 6). Walktrap analysis is a community detection algorithm used to identify densely connected communities within a larger group. The algorithm is based on the idea that random walks within a graph tend to get trapped in densely connected regions, which correspond to communities. Here, walktrap network analysis was performed on the adjacency matrices, resulting in 56 and 52 clusters for the cancer and control samples, respectively. Interestingly, we found a cancer-associated cluster containing three of the six species most strongly related with MSI tumours: *P. micra*, *F. nucleatum*, and *P. stomatis* (Fig. 6a). In addition, this cluster also included several other bacteria previously associated with CRC: *Bacteroides fragilis* and various *Porphyromonas* spp. Moreover, *P. micra*, *B. fragilis*, and *F. nucleatum* were all hubs in the cluster, i.e., they were all connected with more edges than on average in the cluster. Many of the species in the cluster were oral pathogens, including *P. micra*, *F. nucleatum*, *P. stomatis*, as well as *Porphyromonas* species. In the control samples, a cluster containing *P. micra* was also found. However, this cluster differed substantially from the cluster in the cancer group, except for the inclusion of different *Porphyromonas* species, and included fewer taxa (Fig. 6b). A closer analysis of the bacterial species identified within the *P. micra* cancer cluster revealed a significantly higher abundance of *P. micra*, *F. nucleatum*, and *P. stomatis* in faeces from cancer patients compared to controls (Fig. 7).

## Discussion

In this study, we performed the 16S rRNA sequencing of faecal samples from patients with CRC and controls. We found the oral bacteria *P. micra* and *F. nucleatum* to be associated with a cluster of CRC-associated bacteria including several other oral pathogens. This finding aligns with emerging research on the role of oral bacteria in CRC development and progression, suggesting potential pathways for microbial translocation and inflammation [12, 35, 36]. Furthermore, we discovered a significant difference in microbial composition depending on tumour location and MSI status. Interestingly, *P. micra*, *F. nucleatum*, and *P. stomatis*, three of the six annotated species most strongly associated with MSI tumours, were also included in the identified cancer cluster.

A comparison of the faecal microbiomes between CRC patients and controls at the phylum level revealed a higher proportion of *Firmicutes*, and a lower proportion of *Actinobacteria* and *Bacteriodota* in CRC patients. Changes like these in the gut microbial community structure may disrupt the balance of the gut ecosystem, potentially increasing gut permeability, promoting inflammation and causing epithelial damage, all of which may contribute to tumour development and progression. Although analyses at phylum level offer a broad overview of the microbial structure, more detailed information is required to reveal functional implications for health, as a single phylum may contain both beneficial and harmful bacteria for the host. For instance, the phylum *Firmicutes* includes beneficial species that help maintain gut homeostasis by fermenting dietary fiber and producing short-chain fatty acids [37]. However, opportunistic pathogens such as *Parvimonas* and *Peptostreptococcus* are also part of this phylum. Therefore, more detailed analyses of bacterial species and their interactions with other microbes and the host are essential for assessing their impact on gut health and disease progression.

Two out of four alpha-diversity models suggested a significant difference in bacteria between faecal samples from CRC patients and controls, indicating that CRC patients had a more diverse microbiota than did controls. There are conflicting literature reports on the alpha diversity of gut microbiota in cancer patients and healthy individuals. Most commonly, the alpha diversity of the intestinal microbiota is reported to be significantly lower in CRC patients than in healthy control groups [38–41]. However, there are also contradictory studies reporting higher diversity in CRC patients [42, 43]. Thomas et al. found the gut microbiome in CRC patients to have greater richness than in healthy individuals, partly due to the presence of oral bacteria rarely found in the healthy gut, challenging the common assumption that decreased alpha diversity is generally associated with intestinal dysbiosis [44]. Even though a large proportion of gut microbiota studies are based on 16S rRNA sequencing for gut microbiome analysis, there are differences in sampling, analysis methods, targeted variable regions, sequencing platforms, and databases for taxonomic assignment. Moreover, low sequencing depth can lead to loss of rare species, affecting the outcome. The continuous development of better analysis pipelines is needed.

Furthermore, beta diversity analysis revealed a significant disparity in bacterial composition in faeces between CRC patients and controls. Further analysis using XGBoost resulted in a model that could predict CRC with an AUC of 0.99. The most important taxa for the prediction model were largely in line with previous studies of the microbial segregation between CRC patients and healthy individuals, although the importance of each taxon varied across datasets [4, 32–34]. ASVs that were found differentially abundant between CRC patients and controls included the *Christensenellaceae* R7 group, *Porphyromonsas spp*, and *Prevotella* spp. The *Christensenellaceae* R7 group has previously been suggested to be part of the normal flora, and the importance of this bacterial family in the CRC context is unknown [45]. *Porphyromonas gingivalis* has been associated with tumourigenesis in CRC through its production of butyrate and activation of the NLRP3 inflammasome, and has been linked to poorer patient prognosis [46–48]. *P. gingivalis* has further been shown to promote biofilm formation by *F. nucleatum* [49]. A synergistic effect of *Prevotella intermedica* and *F. nucleatum* in CRC tumourigenesis has also been reported [50]. Interestingly, most of these bacterial species are also found in the oral cavity, highlighting the importance of oral pathogens in CRC, for which periodontal disease is a risk factor [51]. Different possible underlying mechanisms have been suggested, including translocation of bacteria from the oral cavity to the gut and an increase of systemic inflammation and immune dysregulation in periodontitis, leading to an altered gut microbiota [12, 35, 52]. However, the exact mechanisms remain to be further explored.

To find possible differences in faecal bacterial composition within CRC patients, beta diversity analyses were conducted in relation to clinicopathological and molecular features. The results indicated a significant difference in microbial composition depending on tumour MSI status and location, which has also been suggested in previous studies [53, 54]. Subsequent analysis revealed an abundance in MSI tumours of bacteria previously associated with CRC [3, 55]. *F. nucleatum* has been extensively studied and has been associated with MSI tumours in various studies [29, 56, 57]. In our previous studies, we found both *P. micra* and *F. nucleatum* to be more abundant in patients with MSI tumours. [14, 15] Purcell et al. found *P. micra*, *F. nucleatum*, and *P. stomatis* to be enriched in the tumour consensus molecular subtype 1 (CMS1), which mainly includes tumours of the MSI subtype. Together, our findings suggest that at least *P. micra*, *F. nucleatum*, and *P. stomatis* could be associated with MSI tumours. More studies are needed to confirm an association for the other species found to be associated with MSI in this study. MSI tumours are recognised by high immunogenicity and immune infiltration [58, 59] Consequently, MSI has been approved as a predictive marker for immunotherapy in CRC. Microbial shifts have been linked to tumour-initiating inflammation, [2] and response to both chemotherapy and immunotherapy in CRC [60, 61]. For instance, *F. nucleatum* has been shown to promote M2 macrophage polarisation resulting in a decreased infiltration of CD3^+^ T cells [62, 63]. In contrast, we have previously linked *P. micra* to increased immune activation [64]. CRC patients with MSI tumours generally have improved survival [65]. However, bacteria such as *F. nucleatum* and *P. micra* have been associated with decreased survival in CRC patients, [11, 27–29, 66] suggesting that these bacteria may identify a subgroup of MSI tumours with a poorer prognosis. The interaction of bacteria with MSI tumours may additionally affect the response to therapy. Learning more about the specific implications of individual bacteria and their interactions in CRC, will likely contribute to important therapeutic improvements.

Since MSI tumours are more common in right-sided CRCs and a significant difference in beta diversity was found for tumour location, further analysis was performed comparing bacteria in the right colon versus the left colon and rectum. However, the species found to be associated with MSI tumours were not differently abundant according to tumour location. Moreover, the bacteria found to be associated with right-sided tumours were largely unknown in the CRC context and need to be explored further.

Next, we wanted to investigate the correlations between different CRC-associated bacteria, with a main focus on the oral pathogens *P. micra* and *F. nucleatum*. In previous studies using specific qPCR assays, we have found *P. micra* and *F. nucleatum* to have similar distributions in both faecal and tumour tissue samples from patients with CRC [13–15]. This association was also found by Yu et al. in faeces [67]. While *P. micra*’s role in CRC is less explored, *F. nucleatum* is one of the best-studied bacteria in relation to CRC and has been closely associated with CRC progression [3–8, 67]. *F. nucleatum* has also been associated with important clinical and molecular tumour features, including MSI tumours, right-sided tumour location, reduced chemotherapy response, and decreased survival [27–30]. In this study, ASVs annotated to *F. nucleatum* did not cluster together using the LULU algorithm and thus did not pass the filtering process. However, given the significant interest in *F. nucleatum* in the CRC context, and to ensure comparability with earlier studies, all ASVs annotated to *F. nucleatum* were manually clustered together and included in subsequent analyses. The taxonomy of *Fusobacterium* underwent recent revision, elevating subspp. *animalis* and *vincentii* to the rank of species [22]. A potential reason for the lack of clustering among ASVs annotated to *F. nucleatum* could thus be due to the significant genetic diversity among these organisms, prompting the revision [31]. Further studies of the importance of the different *Fusobacterium* taxonomic entities are needed, including the new *F. animalis* and *F. vincentii* species.

Using network analysis, we found a cluster of bacteria in the faeces of CRC patients containing both *P. micra* and *F. nucleatum* as well as several other bacteria, such as the oral pathogens *P. stomatis*, one of the most important taxa in our CRC prediction model, and various *Porphyromonas* species. The cluster also included *B. fragilis*, which has been suggested to contribute to CRC development through toxin production [68]. Both *P*. *micra* and *F. nucleatum*, as well as *B*. *fragilis*, were hubs in the cluster, highlighting their importance for the cluster formation. Thus, despite the risks of false positives and unknown false negatives, this cluster included several bacteria known to be associated with CRC. Also, *P. micra*, *F. nucleatum* and *P. stomatis* were found more highly abundant in faeces of CRC patients compared to controls, suggesting a potential role in CRC progression. In the faeces of the controls, a cluster containing *P. micra*, but not *F. nucleatum*, was found. This cluster differed substantially from the cluster in the cancer group. Our findings are corroborated in a recent study by Conde-Pèrez et al., where they describe a similar cluster of oral pathobionts in CRC containing *Fusobacterium*, *Parvimonas* and *Peptostreptococcus* genera, along with e.g. *Bacteroides fragilis* [69]. These findings together suggest the potential value of identifying bacterial clusters for both screening and treatment of CRC.

This study does not provide any mechanistic insights for the found associations. Thus, further investigations into the biological pathways through which *P. micra* and interacting bacteria might influence CRC development and progression are necessary to improve our understanding of the causal relationship of the gut microbial composition in CRC. Another limitation of this study is that our findings related to the microbiome are restricted to the time of disease diagnosis. Longitudinal stool sampling throughout disease development and progression would greatly enhance our understanding of the role of the intestinal microbiome in CRC. However, we do believe that our study design, with stool samples being collected by the patients in their home in close vicinity to diagnosis and before start of treatment, contributes important findings. The lack of data on confounding environmental and health factors also limits our study. Further studies are needed exploring the influences of environmental and health factors on the microbiome and their possible role in CRC development and progression. The study sample size and lack of validation cohort pose further limitations. Even though the findings of this study represent a significant step in understanding CRC within this specific population, we recognize that genetic, environmental, and lifestyle factors may vary across different populations and encourage the replication of our findings in more diverse populations.

## Conclusions

Our study reveals distinct differences in the faecal microbiome between CRC patients and controls. Bacterial variations were found based on tumour location and MSI status. Notably, the CRC-associated oral pathogens *P. micra* and *F. nucleatum* were found alongside other relevant bacteria, suggesting potential collaborations between bacteria within the tumour microenvironment. Our findings underscore the need for further studies to deepen our understanding of these interactions and their impact on CRC development and progression, potentially guiding biomarker discovery and future therapeutic strategies for CRC.

## Electronic supplementary material

Below is the link to the electronic supplementary material.

Supplementary Material 1

Supplementary Material 2

## Data Availability

Data is available from the corresponding author on a reasonable request. Code for the LULU implementation is available at https://github.com/Clinical-Genomics-Umeå/nf_16S_metagen and for the statistical analyses at https://github.com/Clinical-Genomics-Umeå/nextflow_16S_metagenomic_RIP.

## References

[1] Wong SH, Yu J. Gut microbiota in colorectal cancer: mechanisms of action and clinical applications. Nat Rev Gastroenterol Hepatol. 2019;16(11):690–704. 31554963 10.1038/s41575-019-0209-8

[2] Tjalsma H, Boleij A, Marchesi JR, Dutilh BE. A bacterial driver-passenger model for colorectal cancer: beyond the usual suspects. Nat Rev Microbiol. 2012;10(8):575–82. 22728587 10.1038/nrmicro2819

[3] Dai Z, Coker OO, Nakatsu G, Wu WKK, Zhao L, Chen Z, et al. Multi-cohort analysis of colorectal cancer metagenome identified altered bacteria across populations and universal bacterial markers. Microbiome. 2018;6(1):70. 29642940 10.1186/s40168-018-0451-2 PMC5896039

[4] Wirbel J, Pyl PT, Kartal E, Zych K, Kashani A, Milanese A, et al. Meta-analysis of fecal metagenomes reveals global microbial signatures that are specific for colorectal cancer. Nat Med. 2019;25(4):679–89. 30936547 10.1038/s41591-019-0406-6 PMC7984229

[5] Baxter NT, Ruffin MTt, Rogers MA, Schloss PD. Microbiota-based model improves the sensitivity of fecal immunochemical test for detecting colonic lesions. Genome Med. 2016;8(1):37. 27056827 10.1186/s13073-016-0290-3 PMC4823848

[6] Drewes JL, White JR, Dejea CM, Fathi P, Iyadorai T, Vadivelu J, et al. High-resolution bacterial 16S rRNA gene profile meta-analysis and biofilm status reveal common colorectal cancer consortia. NPJ Biofilms Microbiomes. 2017;3:34. 29214046 10.1038/s41522-017-0040-3 PMC5707393

[7] Kostic AD, Chun E, Robertson L, Glickman JN, Gallini CA, Michaud M, et al. Fusobacterium nucleatum potentiates intestinal tumorigenesis and modulates the tumor-immune microenvironment. Cell Host Microbe. 2013;14(2):207–15. 23954159 10.1016/j.chom.2013.07.007 PMC3772512

[8] Wong SH, Kwong TNY, Chow TC, Luk AKC, Dai RZW, Nakatsu G, et al. Quantitation of faecal Fusobacterium improves faecal immunochemical test in detecting advanced colorectal neoplasia. Gut. 2017;66(8):1441–8. 27797940 10.1136/gutjnl-2016-312766 PMC5530471

[9] Rubinstein MR, Wang X, Liu W, Hao Y, Cai G, Han YW. Fusobacterium nucleatum promotes colorectal carcinogenesis by modulating E-cadherin/β-catenin signaling via its FadA adhesin. Cell Host Microbe. 2013;14(2):195–206. 23954158 10.1016/j.chom.2013.07.012 PMC3770529

[10] Shah MS, DeSantis TZ, Weinmaier T, McMurdie PJ, Cope JL, Altrichter A, et al. Leveraging sequence-based faecal microbial community survey data to identify a composite biomarker for colorectal cancer. Gut. 2018;67(5):882–91. 28341746 10.1136/gutjnl-2016-313189

[11] Zhao L, Zhang X, Zhou Y, Fu K, Lau HC, Chun TW, et al. Parvimonas micra promotes colorectal tumorigenesis and is associated with prognosis of colorectal cancer patients. Oncogene. 2022;41(36):4200–10. 35882981 10.1038/s41388-022-02395-7 PMC9439953

[12] Mo S, Ru H, Huang M, Cheng L, Mo X, Yan L. Oral-intestinal microbiota in Colorectal Cancer: inflammation and immunosuppression. J Inflamm Res. 2022;15:747–59. 35153499 10.2147/JIR.S344321 PMC8824753

[13] Lowenmark T, Lofgren-Burstrom A, Zingmark C, Eklof V, Dahlberg M, Wai SN, et al. Parvimonas micra as a putative non-invasive faecal biomarker for colorectal cancer. Sci Rep. 2020;10(1):15250. 32943695 10.1038/s41598-020-72132-1 PMC7499209

[14] Löwenmark T, Li X, Löfgren-Burström A, Zingmark C, Ling A, Kellgren TG et al. Parvimonas micra is associated with tumour immune profiles in molecular subtypes of colorectal cancer. Cancer Immunol Immunother. 2022. 10.1007/s00262-022-03179-4 PMC9463256 35301576

[15] Löwenmark T, Löfgren-Burström A, Zingmark C, Ljuslinder I, Dahlberg M, Edin S et al. Tumour Colonisation of Parvimonas micra is Associated with decreased survival in Colorectal Cancer patients. Cancers (Basel). 2022;14(23). 10.3390/cancers14235937 PMC9736682 36497419

[16] Horiuchi A, Kokubu E, Warita T, Ishihara K. Synergistic biofilm formation by Parvimonas micra and Fusobacterium nucleatum. Anaerobe. 2020;62:102100. 31521732 10.1016/j.anaerobe.2019.102100

[17] Dejea CM, Wick EC, Hechenbleikner EM, White JR, Mark Welch JL, Rossetti BJ, et al. Microbiota organization is a distinct feature of proximal colorectal cancers. Proc Natl Acad Sci U S A. 2014;111(51):18321–6. 25489084 10.1073/pnas.1406199111 PMC4280621

[18] Eklöf V, Löfgren-Burström A, Zingmark C, Edin S, Larsson P, Karling P, et al. Cancer-associated fecal microbial markers in colorectal cancer detection. Int J Cancer. 2017;141(12):2528–36. 28833079 10.1002/ijc.31011 PMC5697688

[19] Glimelius B, Melin B, Enblad G, Alafuzoff I, Beskow A, Ahlstrom H, et al. U-CAN: a prospective longitudinal collection of biomaterials and clinical information from adult cancer patients in Sweden. Acta Oncol. 2018;57(2):187–94. 28631533 10.1080/0284186X.2017.1337926

[20] Callahan BJ, McMurdie PJ, Rosen MJ, Han AW, Johnson AJ, Holmes SP. DADA2: high-resolution sample inference from Illumina amplicon data. Nat Methods. 2016;13(7):581–3. 27214047 10.1038/nmeth.3869 PMC4927377

[21] Frøslev TG, Kjøller R, Bruun HH, Ejrnæs R, Brunbjerg AK, Pietroni C, et al. Algorithm for post-clustering curation of DNA amplicon data yields reliable biodiversity estimates. Nat Commun. 2017;8(1):1188. 29084957 10.1038/s41467-017-01312-x PMC5662604

[22] Munson E, Carella A, Carroll KC. Valid and accepted novel bacterial taxa derived from human clinical specimens and taxonomic revisions published in 2022. J Clin Microbiol. 2023;61(11):e0083823. 37889007 10.1128/jcm.00838-23 PMC10662342

[23] Li X, Larsson P, Ljuslinder I, Ohlund D, Myte R, Lofgren-Burstrom A et al. Ex vivo organoid cultures reveal the importance of the Tumor Microenvironment for maintenance of Colorectal Cancer Stem cells. Cancers (Basel). 2020;12(4). 10.3390/cancers12040923 PMC7226030 32290033

[24] Dominance statistics: Ordinal analyses to answer ordinal questions. (1993).

[25] Chen T, Guestrin C. XGBoost: a scalable tree boosting System2016. 785 – 94 p.

[26] Pons P, Latapy M, editors. Computing communities in large networks using random walks. Computer and Information Sciences-ISCIS 2005: 20th International Symposium, Istanbul, Turkey, October 26–28, 2005 Proceedings 20; 2005: Springer.

[27] Wei Z, Cao S, Liu S, Yao Z, Sun T, Li Y, et al. Could gut microbiota serve as prognostic biomarker associated with colorectal cancer patients’ survival? A pilot study on relevant mechanism. Oncotarget. 2016;7(29):46158–72. 27323816 10.18632/oncotarget.10064 PMC5216788

[28] Chen Y, Lu Y, Ke Y, Li Y. Prognostic impact of the Fusobacterium nucleatum status in colorectal cancers. Med (Baltim). 2019;98(39):e17221. 10.1097/MD.0000000000017221 PMC6775385 31574832

[29] Mima K, Nishihara R, Qian ZR, Cao Y, Sukawa Y, Nowak JA, et al. Fusobacterium nucleatum in colorectal carcinoma tissue and patient prognosis. Gut. 2016;65(12):1973–80. 26311717 10.1136/gutjnl-2015-310101 PMC4769120

[30] Zhang S, Yang Y, Weng W, Guo B, Cai G, Ma Y, et al. Fusobacterium nucleatum promotes chemoresistance to 5-fluorouracil by upregulation of BIRC3 expression in colorectal cancer. J Exp Clin Cancer Res. 2019;38(1):14. 30630498 10.1186/s13046-018-0985-y PMC6327560

[31] Bi D, Zhu Y, Gao Y, Li H, Zhu X, Wei R, et al. A newly developed PCR-based method revealed distinct Fusobacterium nucleatum subspecies infection patterns in colorectal cancer. Microb Biotechnol. 2021;14(5):2176–86. 34309194 10.1111/1751-7915.13900 PMC8449656

[32] Parker BJ, Wearsch PA, Veloo ACM, Rodriguez-Palacios A. The Genus Alistipes: gut Bacteria with emerging implications to inflammation, Cancer, and Mental Health. Front Immunol. 2020;11. 10.3389/fimmu.2020.00906 PMC7296073 32582143

[33] Wang T, Cai G, Qiu Y, Fei N, Zhang M, Pang X, et al. Structural segregation of gut microbiota between colorectal cancer patients and healthy volunteers. ISME J. 2012;6(2):320–9. 21850056 10.1038/ismej.2011.109 PMC3260502

[34] Hua H, Sun Y, He X, Chen Y, Teng L, Lu C. Intestinal microbiota in colorectal adenoma-carcinoma sequence. Front Med (Lausanne). 2022;9:888340. 35935780 10.3389/fmed.2022.888340 PMC9348271

[35] Ajwani S, Mattila KJ, Narhi TO, Tilvis RS, Ainamo A. Oral health status, C-reactive protein and mortality–a 10 year follow-up study. Gerodontology. 2003;20(1):32–40. 12926749 10.1111/j.1741-2358.2003.00032.x

[36] Conde-Perez K, Buetas E, Aja-Macaya P, Martin-De Arribas E, Iglesias-Corras I, Trigo-Tasende N, et al. Parvimonas micra can translocate from the subgingival sulcus of the human oral cavity to colorectal adenocarcinoma. Mol Oncol. 2024;18(5):1143–73. 37558206 10.1002/1878-0261.13506 PMC11076991

[37] Sun Y, Zhang S, Nie Q, He H, Tan H, Geng F, et al. Gut firmicutes: relationship with dietary fiber and role in host homeostasis. Crit Rev Food Sci Nutr. 2023;63(33):12073–88. 35822206 10.1080/10408398.2022.2098249

[38] Wang WJ, Zhou YL, He J, Feng ZQ, Zhang L, Lai XB, et al. Characterizing the composition of intestinal microflora by 16S rRNA gene sequencing. World J Gastroenterol. 2020;26(6):614–26. 32103871 10.3748/wjg.v26.i6.614 PMC7029349

[39] Yang Y, Misra BB, Liang L, Bi D, Weng W, Wu W, et al. Integrated microbiome and metabolome analysis reveals a novel interplay between commensal bacteria and metabolites in colorectal cancer. Theranostics. 2019;9(14):4101–14. 31281534 10.7150/thno.35186 PMC6592169

[40] Liu W, Zhang R, Shu R, Yu J, Li H, Long H, et al. Study of the relationship between Microbiome and Colorectal Cancer susceptibility using 16SrRNA sequencing. Biomed Res Int. 2020;2020:7828392. 32083132 10.1155/2020/7828392 PMC7011317

[41] Ahn J, Sinha R, Pei Z, Dominianni C, Wu J, Shi J, et al. Human gut microbiome and risk for colorectal cancer. J Natl Cancer Inst. 2013;105(24):1907–11. 24316595 10.1093/jnci/djt300 PMC3866154

[42] Thomas AM, Jesus EC, Lopes A, Aguiar S Jr., Begnami MD, Rocha RM, et al. Tissue-Associated bacterial alterations in rectal carcinoma patients revealed by 16S rRNA community profiling. Front Cell Infect Microbiol. 2016;6:179. 28018861 10.3389/fcimb.2016.00179 PMC5145865

[43] Mira-Pascual L, Cabrera-Rubio R, Ocon S, Costales P, Parra A, Suarez A, et al. Microbial mucosal colonic shifts associated with the development of colorectal cancer reveal the presence of different bacterial and archaeal biomarkers. J Gastroenterol. 2015;50(2):167–79. 24811328 10.1007/s00535-014-0963-x

[44] Thomas AM, Manghi P, Asnicar F, Pasolli E, Armanini F, Zolfo M, et al. Metagenomic analysis of colorectal cancer datasets identifies cross-cohort microbial diagnostic signatures and a link with choline degradation. Nat Med. 2019;25(4):667–78. 30936548 10.1038/s41591-019-0405-7 PMC9533319

[45] Mancabelli L, Milani C, Lugli GA, Turroni F, Cocconi D, van Sinderen D et al. Identification of universal gut microbial biomarkers of common human intestinal diseases by meta-analysis. FEMS Microbiol Ecol. 2017;93(12). 10.1093/femsec/fix153 29126267

[46] Kerdreux M, Edin S, Lowenmark T, Bronnec V, Lofgren-Burstrom A, Zingmark C, et al. Porphyromonas gingivalis in Colorectal Cancer and its Association to Patient Prognosis. J Cancer. 2023;14(9):1479–85. 37325051 10.7150/jca.83395 PMC10266249

[47] Okumura S, Konishi Y, Narukawa M, Sugiura Y, Yoshimoto S, Arai Y, et al. Gut bacteria identified in colorectal cancer patients promote tumourigenesis via butyrate secretion. Nat Commun. 2021;12(1):5674. 34584098 10.1038/s41467-021-25965-x PMC8479117

[48] Wang X, Jia Y, Wen L, Mu W, Wu X, Liu T, et al. Porphyromonas gingivalis promotes colorectal carcinoma by activating the hematopoietic NLRP3 Inflammasome. Cancer Res. 2021;81(10):2745–59. 34003774 10.1158/0008-5472.CAN-20-3827

[49] Saito Y, Fujii R, Nakagawa KI, Kuramitsu HK, Okuda K, Ishihara K. Stimulation of Fusobacterium nucleatum biofilm formation by Porphyromonas gingivalis. Oral Microbiol Immunol. 2008;23(1):1–6. 18173791 10.1111/j.1399-302X.2007.00380.x

[50] Lo CH, Wu DC, Jao SW, Wu CC, Lin CY, Chuang CH, et al. Enrichment of Prevotella intermedia in human colorectal cancer and its additive effects with Fusobacterium nucleatum on the malignant transformation of colorectal adenomas. J Biomed Sci. 2022;29(1):88. 36303164 10.1186/s12929-022-00869-0 PMC9615364

[51] Momen-Heravi F, Babic A, Tworoger SS, Zhang L, Wu K, Smith-Warner SA, et al. Periodontal disease, tooth loss and colorectal cancer risk: results from the nurses’ Health Study. Int J Cancer. 2017;140(3):646–52. 27778343 10.1002/ijc.30486 PMC5159274

[52] Conde-Pérez K, Buetas E, Aja-Macaya P, Arribas EM, Iglesias-Corrás I, Trigo-Tasende N et al. Parvimonas micra can translocate from the subgingival sulcus of the human oral cavity to colorectal adenocarcinoma. Mol Oncol. 2023. 10.1002/1878-0261.13506 PMC11076991 37558206

[53] Kneis B, Wirtz S, Weber K, Denz A, Gittler M, Geppert C et al. Colon Cancer Microbiome Landscaping: differences in right- and left-sided Colon cancer and a Tumor Microbiome-Ileal Microbiome Association. Int J Mol Sci. 2023;24(4). 10.3390/ijms24043265 PMC9963782 36834671

[54] Phipps O, Quraishi MN, Dickson EA, Steed H, Kumar A, Acheson AG et al. Differences in the On- and off-tumor microbiota between right- and left-sided colorectal Cancer. Microorganisms. 2021;9(5). 10.3390/microorganisms9051108 PMC8160982 34065545

[55] Yang Y, Du L, Shi D, Kong C, Liu J, Liu G, et al. Dysbiosis of human gut microbiome in young-onset colorectal cancer. Nat Commun. 2021;12(1):6757. 34799562 10.1038/s41467-021-27112-y PMC8604900

[56] Tahara T, Yamamoto E, Suzuki H, Maruyama R, Chung W, Garriga J, et al. Fusobacterium in colonic flora and molecular features of colorectal carcinoma. Cancer Res. 2014;74(5):1311–8. 24385213 10.1158/0008-5472.CAN-13-1865 PMC4396185

[57] Ito M, Kanno S, Nosho K, Sukawa Y, Mitsuhashi K, Kurihara H, et al. Association of Fusobacterium nucleatum with clinical and molecular features in colorectal serrated pathway. Int J Cancer. 2015;137(6):1258–68. 25703934 10.1002/ijc.29488

[58] Deschoolmeester V, Baay M, Lardon F, Pauwels P, Peeters M. Immune cells in Colorectal Cancer: Prognostic Relevance and Role of MSI. Cancer Microenviron. 2011;4(3):377–92. 21618031 10.1007/s12307-011-0068-5 PMC3234325

[59] Randrian V, Evrard C, Tougeron D. Microsatellite instability in colorectal cancers: Carcinogenesis, Neo-antigens, Immuno-Resistance and emerging therapies. Cancers (Basel). 2021;13(12). 10.3390/cancers13123063 PMC8235567 34205397

[60] Routy B, Le Chatelier E, Derosa L, Duong CPM, Alou MT, Daillere R, et al. Gut microbiome influences efficacy of PD-1-based immunotherapy against epithelial tumors. Science. 2018;359(6371):91–7. 29097494 10.1126/science.aan3706

[61] Yu T, Guo F, Yu Y, Sun T, Ma D, Han J, et al. Fusobacterium nucleatum promotes Chemoresistance to Colorectal Cancer by modulating Autophagy. Cell. 2017;170(3):548–63. e16. 28753429 10.1016/j.cell.2017.07.008 PMC5767127

[62] Chen T, Li Q, Wu J, Wu Y, Peng W, Li H, et al. Fusobacterium nucleatum promotes M2 polarization of macrophages in the microenvironment of colorectal tumours via a TLR4-dependent mechanism. Cancer Immunol Immunother. 2018;67(10):1635–46. 30121899 10.1007/s00262-018-2233-x PMC11028377

[63] Mima K, Cao Y, Chan AT, Qian ZR, Nowak JA, Masugi Y, et al. Fusobacterium nucleatum in Colorectal Carcinoma tissue according to Tumor Location. Clin Transl Gastroenterol. 2016;7(11):e200. 27811909 10.1038/ctg.2016.53 PMC5543402

[64] Löwenmark T, Li X, Löfgren-Burström A, Zingmark C, Ling A, Kellgren TG, et al. Parvimonas micra is associated with tumour immune profiles in molecular subtypes of colorectal cancer. Immunotherapy: Cancer Immunology; 2022. 10.1007/s00262-022-03179-4 PMC9463256 35301576

[65] Taieb J, Svrcek M, Cohen R, Basile D, Tougeron D, Phelip JM. Deficient mismatch repair/microsatellite unstable colorectal cancer: diagnosis, prognosis and treatment. Eur J Cancer. 2022;175:136–57. 36115290 10.1016/j.ejca.2022.07.020

[66] Lowenmark T, Lofgren-Burstrom A, Zingmark C, Ljuslinder I, Dahlberg M, Edin S et al. Tumour Colonisation of Parvimonas micra is Associated with decreased survival in Colorectal Cancer patients. Cancers (Basel). 2022;14(23). 10.3390/cancers14235937 PMC9736682 36497419

[67] Yu J, Feng Q, Wong SH, Zhang D, Liang QY, Qin Y, et al. Metagenomic analysis of faecal microbiome as a tool towards targeted non-invasive biomarkers for colorectal cancer. Gut. 2017;66(1):70–8. 26408641 10.1136/gutjnl-2015-309800

[68] Toprak NU, Yagci A, Gulluoglu BM, Akin ML, Demirkalem P, Celenk T, et al. A possible role of Bacteroides fragilis enterotoxin in the aetiology of colorectal cancer. Clin Microbiol Infect. 2006;12(8):782–6. 16842574 10.1111/j.1469-0691.2006.01494.x

[69] Conde-Perez K, Aja-Macaya P, Buetas E, Trigo-Tasende N, Nasser-Ali M, Rumbo-Feal S, et al. The multispecies microbial cluster of Fusobacterium, Parvimonas, Bacteroides and Faecalibacterium as a precision biomarker for colorectal cancer diagnosis. Mol Oncol. 2024;18(5):1093–122. 38366793 10.1002/1878-0261.13604 PMC11076999

