# Supplementary material for: Parvimonas micra forms a distinct bacterial network with oral pathobionts in colorectal cancer patients
Source: J Transl Med. 2024 Oct 17;22:947. doi: 10.1186/s12967-024-05720-8 (PMC11487773; doi:10.1186/s12967-024-05720-8)
Supplement: Supplementary file 1 — Supplementary Material 1 [file 12967_2024_5720_MOESM1_ESM.pdf]

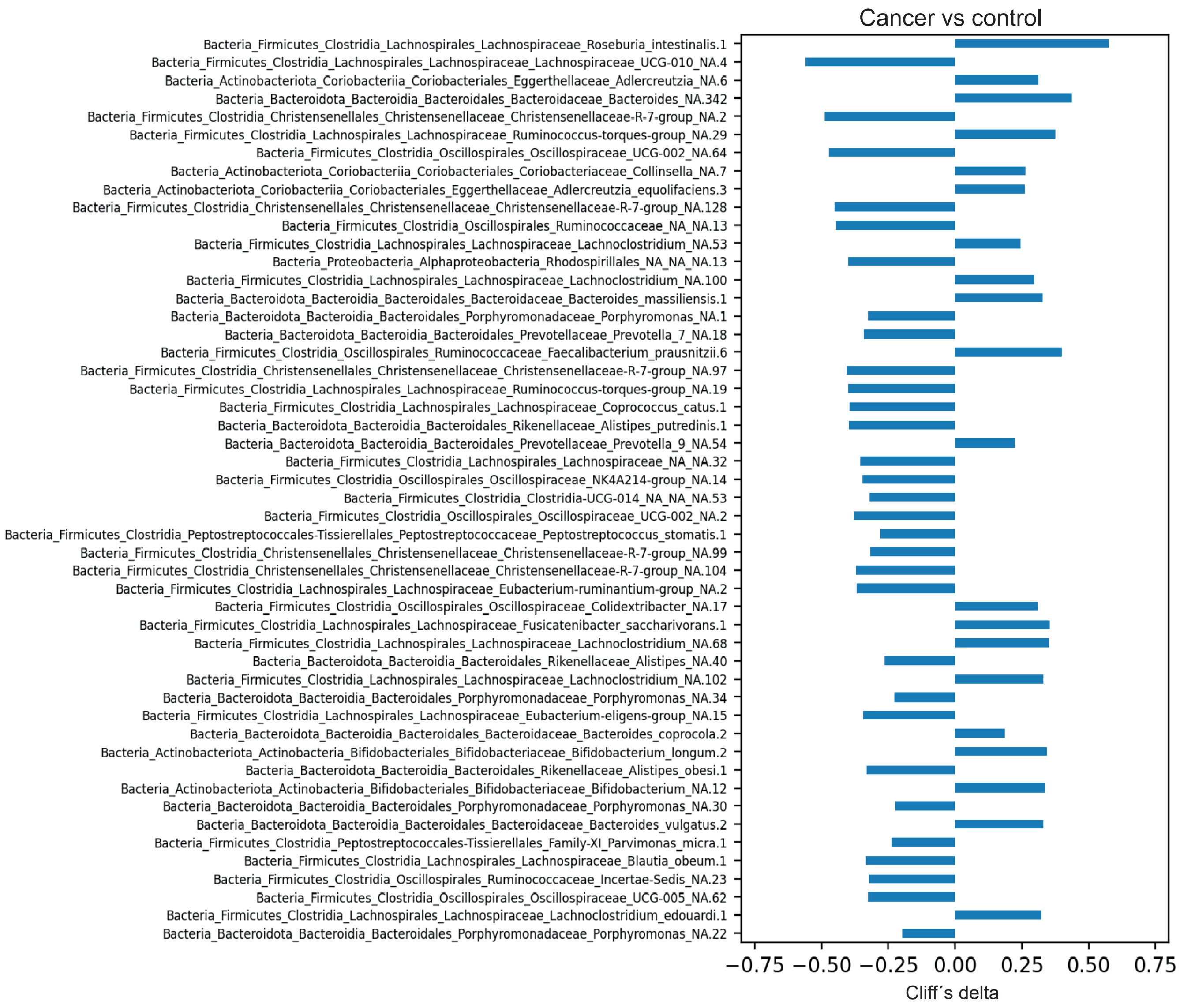

Figure S1. Top ASVs with most significant differences in abundance between cancer and control groups as determined by the MWU test. ASVs were sorted according to p-value, displaying the ASV with the lowest p-value at the top (nominal p-values were used). A negative Cliff's delta indicates higher abundance in cancer, whereas a positive Cliff's delta indicates a higher abundance in controls.
